# *De novo* Genome Assessment of Serratia marcescens SGT5.3, a Potential Plant Growth-Promoting Bacterium Isolated from the Surface of Capsicum annuum Fruit

**DOI:** 10.1128/mra.01154-22

**Published:** 2023-01-04

**Authors:** Tshifhiwa Paris Mamphogoro, Casper Nyaradzai Kamutando, Martin Makgose Maboko, Olayinka Ayobami Aiyegoro, Olubukola Oluranti Babalola

**Affiliations:** a Gastro-Intestinal Microbiology and Biotechnology Unit, Agricultural Research Council–Animal Production, Irene, Pretoria, South Africa; b Department of Plant Production Sciences and Technologies, University of Zimbabwe, Mount Pleasant, Harare, Zimbabwe; c Department of Crop Sciences, Tshwane University of Technology, Pretoria, South Africa; d Research Unit for Environmental Sciences and Management, North-West University, Potchefstroom, South Africa; e Food Security and Safety Focus Area, Faculty of Natural and Agricultural Sciences, North-West University, Mmabatho, South Africa; University of Arizona

## Abstract

Serratia marcescens SGT5.3, a potential plant growth-promoting strain with a wide range of functions, was isolated from the surface of Capsicum annuum fruit. Here, we report the whole-genome sequence of this bacterium. Gene prediction revealed various functional genes potentially involved in plant growth promotion and development.

## ANNOUNCEMENT

Serratia marcescens is a Gram-negative, facultatively anaerobic, rod-shaped, and non-spore-forming bacterium that is known to be associated with several plant species (1, 2). Its associations with plants were shown in some previous studies as beneficial to plant growth and development (3–5). Some reports have also pointed out the potential of this bacterium for producing industrially relevant enzymes, such as lipases and proteases (1).

SGT5.3 was isolated from the surface of fungicide-treated green sweet pepper (Capsicum annuum) fruits from the Agricultural Research Council Division of Vegetable and Ornamental Plants, South Africa. Fresh mature peppers, grown under open soil conditions, were collected transiently in sterile zip-lock bags (6). Bacterial cells from the surfaces of the fruits were obtained as in reference 2, and morphologically distinct single colonies were purified by repeated streaking onto tryptic soy agar (TSA) medium. Genomic DNA was extracted from an overnight liquid culture on tryptic soy broth (TSB) medium using a Quick-DNA miniprep kit (Zymo Research, Irvine, CA, USA) and amplified using 16S rRNA universal primers (27F and 1492R) (7). The amplicon was sequenced, a similarity analysis was performed using the BLAST server, and the sequence was submitted to GenBank. SGT5.3 had high similarity to S. marcescens JW-CZ2 (GenBank accession number KP903465), consisting of a 1,503-bp partial 16S rRNA gene. JW-CZ2 possesses *pst*C, *pst*S, *pho*U, *ipd*C, *hem*H, and *Bfr* genes, which are all linked to plant growth promotion characteristics (8). Strain SGT5.3 was previously reported to harbor plant growth-promoting traits such as phosphate solubilization and siderophore production (2).

Sequencing libraries were prepared using a NEBNext Ultra II FS DNA library prep kit (New England Biolabs, Ipswich, MA, USA), and whole-genome sequencing of S. marcescens strain SGT5.3 was performed on the Illumina NextSeq 550 platform (2 × 150 bp) at Inqaba Biotechnical Industries, South Africa. A total of 2,136,089 150-bp paired-end reads, representing ~125× genome coverage, were generated. The Illumina-generated sequence reads were quality filtered using FastQC v0.11.5 (9), while removal of sequence adaptors and low-quality reads was performed using Trimmomatic v0.36 (10). The quality-filtered reads were assembled *de novo* using SPAdes v3.13.0 (11). Genome annotation was performed using the Prokaryotic Genome Annotation Pipeline (PGAP) v5.3 (12). Default parameters were used for all software unless otherwise specified. The final assembly yielded a 5,125,491-bp genome, with a mean G+C content of 59.8%, and a total of 188 contigs, with an *N*_50_ value of 251,218 bp, an *L*_50_ value of 5, and a total of 5,019 genes, of which 4,873 were protein-coding genes, 79 were tRNA genes, 6 were rRNA genes (5S rRNA, 16S rRNA, 23S rRNA), and 15 were noncoding RNA (ncRNA) genes. The genome also contained 46 pseudogenes.

Functional annotation detected genes related to growth promotion (*glnH*, *glnG*, *nirB*, *nirD*, and *narK*), IAA phytohormones (*trpA*, *trpB*, *trpCF*, and *trpD*), cytokinins (*mia*A and *mia*B), and a siderophore (*entS*, *entF*, *feoB*, *fetB*, and *fhuA*), among others; these genes are likely to be associated with plant growth promotion and development (13–15). The findings demonstrate the genetic capability of strain SGT5.3 for bioprospecting applications in agriculture.

### Data availability.

This whole-genome shotgun project and the associated data have been deposited at DDBJ/ENA/GenBank under the accession number JAJFEW000000000, with the BioProject accession number PRJNA771517, BioSample accession number SAMN22357973, and SRA accession number SRR16379969. The version described in this paper is version JAJFEW000000000.
